# Synergistic effects of multi-enzyme supplementation on nutrient digestion and absorption in the foregut and hindgut

**DOI:** 10.3389/fvets.2025.1554919

**Published:** 2025-02-25

**Authors:** Fangyuan Chen, Lianpeng Zhao, Lingjie Huang, Yong Zhuo, Shengyu Xu, Yan Lin, Lianqiang Che, Bin Feng, De Wu, Zhengfeng Fang

**Affiliations:** ^1^Key Laboratory for Animal Disease Resistance Nutrition of the Ministry of Education, Animal Nutrition Institute, Sichuan Agricultural University, Chengdu, Sichuan, China; ^2^College of Food Science, Sichuan Agricultural University, Yaan, Sichuan, China

**Keywords:** enzyme, growing pig, enzymatic hydrolysis, microbial fermentation, nutrient utilization

## Abstract

This study was conducted to investigate the effect of dietary multi-enzyme (MCPC) supplementation on synergistically enhancing the functions of both the foregut and hindgut, ultimately improving the nutrient digestion and utilization throughout the gastrointestinal tract. *In vitro* results demonstrated that MCPC increased the phosphorus and reducing sugar levels in the supernatant during enzymatic hydrolysis. Furthermore, during the fermentation of the enzymatic hydrolysis products, MCPC significantly increased the FRD_0_ value of the enzymatic hydrolysis products from both the positive control (PC) and negative control 1 (NC1) diets (*p* < 0.05). MCPC reduced the T_1/2_ value of *in vitro* fermentation products from the PC diet (*p* < 0.01), and decreased the V_F_ (*p* = 0.082) and K (*p* < 0.05) values for the NC1 diet. Additionally, 72 crossbred barrows [Duroc × (Landrace × Yorkshire)], weighing 25 kg, were fed one of six diets until their live weight approached 50 kg. The basal diets consisted of PC, NC1 and negative control 2 (NC2), while the remaining three diets were prepared by adding 100 mg/kg MCPC to the respective basal diets. The results showed that MCPC supplementation significantly upregulated the expression of solute carrier family 17 member 4 (*SLC17A4*) and vitamin D receptor (*VDR*) genes in the duodenum (*p* < 0.05), while downregulating the expression of Calbindin-D28k (*CaBP-D28K*) and solute carrier family 1 member 4 (*SLC1A4*) genes (*p* < 0.05) in growing pigs. Moreover, MCPC supplementation significantly upregulated the expression of *VDR*, glucose transporter 2 (*GLUT2*) and intestinal fatty acid binding protein (*FABP2*) genes in the jejunum of growing pigs. Furthermore, MCPC supplementation significantly increased the relative abundances of *Bacteroidota*, *Prevotella* and *Phascolarctobacterium* (*p* < 0.05), while reducing the relative abundances of *Verrucomicrobiota* and *Clostridium_sensu_stricto_1* (*p* < 0.05) in the colon of growing pigs. In conclusion, MCPC enhances nutrient digestion and absorption in the foregut, provides fermentable substrates for hindgut microbial fermentation, and improves gut microbiota composition. This improves hindgut fermentation and supports the synergistic interaction between the foregut and hindgut, ultimately improving nutrient utilization and benefiting animal health.

## 1 Introduction

China is a prominent global feed producer, yet feed resources remain relatively scarce. Optimizing feed formulations is critical for enhancing feed efficiency and promoting sustainable livestock production. In pig production, reducing the inclusion of scarce ingredients, such as corn and soybean meal, leads to higher fiber content in the diet. This increase in fiber results in decreased nutrient digestibility and utilization, as well as an elevation in the composition and concentration of anti-nutritional factors (1, 2). Anti-nutritional factors are widespread in pig diets and significantly limit the efficiency of nutrient utilization. Elevated levels of phytic acid and non-starch polysaccharides (NSP) in feed hinder nutrient chelation and the absorption of essential nutrients (3, 4). However, pigs have a limited capacity to enzymatically release phosphorus from phytates and oligosaccharides from NSP. As a consequence, nutrient digestion, absorption, and utilization are compromised, negatively impacting animal health and productivity (3, 5). Thus, enhancing feed utilization is vital for pig production, aiding in resource conservation and environmental protection.

Given the complexity, concentration, and distribution of anti-nutritional factors, the use of exogenous enzymes is often implemented in practical production to enhance feed efficiency. These enzymes help compensate for the limited secretion of both digestive and non-digestive endogenous enzymes in animals (6, 7). Research has demonstrated that supplementing NSP enzymes breaks down polysaccharides, reduces digesta viscosity, and improves nutrient digestibility (8). Similarly, the addition of phytase to the diet not only promotes the release of phosphorus from phytates but also assists in mineral deposition in bones, which is crucial for proper pig growth (9). However, the effects of single-enzyme supplementation on pig performance across different feed formulations are inconsistent (10, 11). Due to the specificity of enzymes for their substrates and the complex, variable nutritional composition of feed, especially with changes in animal growth stages and production conditions, multi-enzyme combinations targeting anti-nutritional factors are considered more effective and economically valuable than single-enzyme supplementation in practical applications (12, 13).

The foregut of the pig is primarily responsible for enzymatic digestion and is a key organ for nutrient absorption. A previous study indicated that the addition of xylanase to feed improved the apparent ileal digestibility (AID) of amino acids and crude protein (CP) in growing pigs (14). Phytase alone has been shown to enhance the expression of intestinal calcium (Ca), phosphorus (P), and oligopeptide transporters, thereby supporting nutrient absorption (5). However, further investigation is needed to determine whether multi-enzyme supplementation (NSP enzyme and phytase) can effectively facilitate substrate breakdown in the foregut and improve nutrient utilization by upregulating the expression of intestinal nutrient transporter genes. In contrast to the foregut, the hindgut primarily relies on microbial fermentation. Nutrients that are not digested and absorbed in the foregut, particularly fiber, are fermented by hindgut microbes, offering additional absorbable nutrients for the host. This fermentation process can also affect microbial composition, contributing to gut health (15, 16). A previous study indicated that phytase or NSP enzymes alone can affect the composition of gut microbiota (17). However, limited studies have examined the combined effects of phytase and NSP enzymes on hindgut microbial populations and their impact on microbial fermentation. Therefore, we hypothesize that dietary multi-enzyme supplementation could enhance nutrient digestion and absorption in the foregut, provide fermentable substrates for hindgut microbial fermentation, and optimize the composition of the gut microbiota, thereby promoting the synergistic interaction between the foregut and hindgut. To test this hypothesis, the present study first investigated the *in vitro* enzymatic hydrolysis of various diets in the foregut using NSP enzymes and phytase complex (MCPC). The diets were formulated to replicate different nutrient levels commonly encountered in practical pig farming, especially under conditions of limited feed resources or higher fiber content. Following this, the fermentation kinetics and final products of the foregut hydrolysis products were assessed through fecal fermentation *in vitro*. Based on the results of the *in vitro* studies, further validation was carried out in growing pigs. The objective was to assess whether MCPC could synergistically enhance the functions of both the foregut and hindgut, thereby improving nutrient intake and utilization to achieve a synergistic effect. The findings of this study may provide valuable insights into enhancing nutrient utilization efficiency in modern pig farming.

## 2 Materials and methods

### 2.1 Ethics approval

The protocol of this study was approved by the Animal Care and Use Committee of the Animal Nutrition Institute, Sichuan Agricultural University (S20174302), and was carried out in accordance with the guidelines provided by the National Research Council for the Care and Use of Laboratory Animals.

### 2.2 *In vitro* enzymatic hydrolysis

A two-step gastric-intestinal method was utilized to evaluate the *in vitro* enzymatic hydrolysis of diets with both normal and reduced nutrient levels, including positive control (PC), negative control 1 (NC1), and negative control 2 (NC2), with and without enzyme supplementation. The experiment was divided into two treatment groups: the control group (CON), which did not receive MCPC supplementation, and the MCPC group, which was supplemented with MCPC during the *in vitro* hydrolysis of the three basal diets. Four replicates were used for each treatment group, with one digestion tube per replicate. MCPC was supplied by Adisseo France SAS (Rovabio, Advance Phy) and supplemented at 100 mg/kg of diet, providing 1800 U/kg xylanase, 1,244 U/kg *β*-glucanase, 6,600 U/kg arabinofuranosidase, and 1,000 FTU phytase in the diets. The ingredients and nutritional composition of the diets are presented in Table 1.

**Table 1:** Ingredients and nutrient levels of the basal diets (%, as-fed basis).

Item	PC	NC1	NC2
Ingredients			
Corn	58.91	56.41	52.10
Soybean meal (CP 46%)	14.10	12.43	11.61
Wheat bran	10.00	15.00	20.00
Wheat (CP 11%)	10.00	10.00	10.00
Soybean hulls	0.90	1.20	1.32
Soybean oil	2.57	1.90	1.97
Limestone	1.15	1.08	1.07
Monocalcium phosphate	0.96	0.59	0.58
L-Lysine HCl (98%)	0.53	0.52	0.51
DL-Methionine (99%)	0.11	0.10	0.09
L-Threonine	0.18	0.17	0.17
L-Tryptophane	0.03	0.03	0.02
L-Valine	0.08	0.07	0.05
NaCl	0.23	0.23	0.23
Premix^a^	0.27	0.27	0.27
Total	100	100	100
Nutritional levels (calculated values)			
Crude protein, %	14.56	14.40	14.41
Crude fat, %	5.50	4.89	4.96
Crude fiber, %	3.62	4.00	4.36
Net energy, kcal/kg	2,475	2,401	2,351
Digestible lysine, %	0.98	0.95	0.93
Digestible methionine, %	0.32	0.31	0.30
Digestible methionine + cysteine, %	0.55	0.53	0.52
Digestible threonine, %	0.59	0.57	0.56
Digestible tryptophan, %	0.17	0.17	0.16
Digestible arginine, %	0.78	0.76	0.76
Digestible histidine, %	0.34	0.33	0.33
Digestible isoleucine, %	0.51	0.49	0.48
Digestible leucine, %	1.17	1.13	1.10
Digestible phenylalanine, %	0.62	0.60	0.59
Digestible valine, %	0.64	0.62	0.61
Total digestible AA, %	6.67	6.46	6.34
Calcium, %	0.66	0.59	0.59
Total phosphorus, %	0.58	0.53	0.56
STTD-P, %	0.31	0.23	0.23

a Provided per kg of diet: vitamin premix 300 mg (vitamin A, 13500 IU; vitamin D_3_, 2,550 IU; vitamin E, 25 mg; vitamin K_3_, 3 mg; vitamin B_1_, 2.4 mg; vitamin B_2_, 6 mg; niacin, 30 mg; d-panthothenic acid, 15 mg; vitamin B_6_, 2.4 mg; vitamin B_12_, 30 μg; d-biotin, 150 μg; folic acid, 1.5 mg, choline 400 mg; copper (CuSO_4_∙ 5H_2_O), 8 mg; iron (FeSO_4_∙7 H_2_O), 90 mg; manganese (MnSO_4_), 4 mg; zinc (ZnSO_4_), 90 mg; iodine (Ca(IO_3_)_2_), 0.28 mg; selenium (Na_2_SeO_3_), 0.3 mg; flavor, 200 mg; antioxidant, 100 mg; anti-mold, 500 mg; Oregano oil, 200 mg).

The two-step gastric-intestinal method followed previously described procedure with suitable modifications (18). Simulated gastric digestion: For each sample, 2.0000 ± 0.0005 g was weighed and placed into a pre-weighed 50 mL centrifuge tube. To this, 25 mL of 0.1 mol/L phosphate-buffered saline (PBS, pH = 6.0) was added and thoroughly mixed with the substrate. HCl (1 mol/L) was then added to adjust the pH value to 2.0. Following this, 1 mL of pepsin solution containing 25 mg of pepsin (3,000 NFU/mg, Sigma P7000) and 0.5 mL of chloramphenicol ethanol solution was introduced. The mixture was incubated in a constant temperature shaker at 39°C for 2 h to simulate gastric digestion. Simulated intestinal digestion: After gastric digestion, 10 mL of 0.2 mol/L PBS was added, and the pH value of the digesta was adjusted to 6.8 using 1 mol/L HCl or NaOH. Finally, 2 mL of pancreatic enzyme solution containing 50 mg of porcine trypsin (≥ 4 USP, Sigma No. P-1750) was added. Digestion continued at 39°C in the constant temperature shaker for an additional 4 h. After digestion, both the supernatant and residue were collected.

### 2.3 *In vitro* fermentation

The foregut enzymatic hydrolysis products (residue) were freeze-dried and used as substrates for fermentation. Fecal inoculum was obtained from three healthy DLY [Duroc × (Landrace × Yorkshire)] growing pigs from the teaching and research base of Sichuan Agricultural University. Two weeks prior to fecal collection, all pigs were fed a basal diet that met the nutritional requirements recommended by the NRC (2012). Fresh feces were collected aseptically from the rectum of each pig, immediately sealed and stored in a water bath at 39°C until further processing. The fresh feces were then thoroughly mixed in an anaerobic workstation (Thermo Fisher 1,029, United States), diluted five times with deoxygenated sterile saline at 39°C, and mechanically stirred for 90 s. The mixture was filtered through four layers of sterile gauze to obtain the mixed microbial solution. This solution was quickly transferred to a 39°C water bath, where continuous CO_2_ flow was maintained to preserve anaerobic conditions until use. The composition and formulation of the fermentation basal medium were based on previous studies (19). The medium was prepared within 1 h prior to completing the fecal hydrolysis process.

Fermentation was carried out in 100 mL glass tubes, with 0.2 g of the sample accurately weighed and placed at the bottom of each tube. Each sample contained four replicates, and two blank control tubes were established. Based on preliminary experiments, 30 mL of fecal inoculum and 30 mL of culture medium were quickly added to the fermentation tubes. CO_2_ was introduced to expel air from the fermentation tubes, which were then sealed with stoppers. The initial scale and start time of fermentation were recorded. The tubes were incubated in a water bath at 39°C for 48 h, with shaking at 70 rpm. Gas production was recorded at 0, 3, 6, 9, 12, 15, 18, 21, 24, 30, 36, and 48 h, with corrections made using the blank control tubes. Fermentation liquids were collected at 0 and 48 h and stored at −20°C for later analysis of pH value and short-chain fatty acids (SCFAs).

### 2.4 Enzymatic hydrolysis supernatant analysis

The content of reducing sugars in the supernatant was determined using the 3,5-dinitrosalicylic acid (DNS) method. The enzymatic hydrolysis products and diets were analyzed for crude protein (CP, AOAC method 988.05) and gross energy (GE), while the supernatant was analyzed for calcium (Ca, AOAC method 927.02) and phosphorus (P, AOAC method 965.05). The digestibility calculations for CP and GE were based on the following formulas:


$$\mathrm{Digestibility}\;\left(\%\right)=\frac{\begin{array}{l} \left(\mathrm{Weight of diet}\times \mathrm{Nutrient in diet}\right)-\\ \left(\begin{array}{l} \mathrm{Weight of hydrolysis product}\times \\ \mathrm{Nutrient in hydrolysis product} \end{array}\right) \end{array}}{\mathrm{Weight of diet}\times \mathrm{Nutrient in diet}}\times 100$$

### 2.5 Kinetics of gas production

The gas curves accumulated during *in vitro* fermentation were modeled using the mathematical model proposed by Tan et al. (20), with the cumulative gas production (V) fitted to a biphasic model. The model equation is as follows:


$$V=V_{F}\times \left[1-\exp\left(-K\times T\right)\right]÷\left[1+\exp\left(B-K\times T\right)\right]$$

where V_F_ denotes the final asymptotic gas volume (mL/g DM), K represents the fractional rate of gas production at a specific time (h^−1^), B is the positive shape parameter without dimension.

FRD_0_, the initial fractional rate of diet at *t*-value = 0 (h^−1^):


$${FRD}_{0}=K÷\left[1+\exp\left(B\right)\right]$$

Half-life (T_1/2_): The time taken to produce half of the final gas volume:


$$T_{1/2}=\ln\left[2+\exp\left(B\right)\right]÷K$$

### 2.6 Measurement of pH value

The fermentation liquid was centrifuged at 8000 g for 10 min to separate the supernatant. The pH value of the supernatant was then measured using a Leici PHS-25 high-precision pH meter (21).

### 2.7 Short-chain fatty acid determination

The detection SCFAs was performed based on a method described in a previous study (21). Briefly, 1 mL of fermentation supernatant was combined with 0.2 mL of 25% (w/v) polyphosphoric acid solution and 23.3 μL of 210 mmol/L crotonic acid solution, followed by incubation at 4°C for 30 min. The mixture was then centrifuged at 15,000 r/min for 10 min and the supernatant was collected. This was mixed with chromatography-grade methanol at a 1:3 dilution. After centrifugation at 10,000 r/min for 5 min, the supernatant was filtered through a 0.22 μm filter into a 1.5 mL chromatography vial for analysis. Samples were analyzed using a CP-3800 gas chromatograph (Varian, GC CP3800) equipped with a 10 μL microsyringe, a flame ionization detector, and a capillary column (HP-FFAP, 30 m length, 0.53 mm internal diameter, 1 μm film thickness).

### 2.8 Animal experimental design

A total of 72 castrated male DLY pigs, each with an initial body weight (BW) of approximately 25 kg, were randomly assigned to six treatment groups with similar initial BW. Each treatment group consisted of 12 replicates, with one pig per replicate. The six treatment groups were as follows: PC, PC + MCPC, NC1, NC1 + MCPC, NC2 and NC2 + MCPC. The experiment lasted for 5 weeks, and the pigs were slaughtered when their average body weight reached around 50 kg.

### 2.9 Experimental diets and animal management

All pigs were fed one of six experimental diets. The PC diet met the NRC (2012) recommended standards for NE, SID-AA, STTD-P, and total Ca levels. The NC1 diet reduced NE and SID-AA by 3% in comparison to the PC diet. The NC2 diet reduced NE and SID-AA by 5% compared to the PC diet. Additionally, both NC1 and NC2 diets had a reduction of 0.08% in STTD-P and 0.07% in total Ca compared to the PC diet. The remaining three MCPC diets (PC + MCPC, NC1 + MCPC, and NC2 + MCPC) were supplemented with 100 mg/kg of MCPC, along with 1800 U/kg xylanase, 1,244 U/kg *β*-glucanase, 6,600 U/kg arabinofuranosidase, and 1,000 FTU phytase per diet. The level of MCPC supplementation was based on previous studies (22, 23).

Throughout the study, all pigs had ad libitum access to feed and water. Feeding times were scheduled at 07:30, 14:00, and 20:00, with the pens maintained at a temperature range of 22–25°C. Regular cleaning and disinfection of the pens were conducted. The pigs were vaccinated and dewormed in accordance with the farm’s routine management and immunization protocols. At the end of the study, all 72 pigs were fasted for 12 h before being slaughtered for sampling. Colonic digesta and tissue samples from the duodenum and jejunum, were collected and stored at −80°C for subsequent analysis.

### 2.10 RNA extraction and quantitative real-time PCR

The relative mRNA expression of candidate genes in the duodenum and jejunum of growing pigs was analyzed using RT-qPCR. Total RNA was extracted from the intestinal samples with TRIzol reagent (Invitrogen, California, United States). cDNA was synthesized using the PrimeScript™ RT Reagent Kit (TaKaRa Biotechnology Co., Ltd). RT-qPCR analysis for mRNA expression of the candidate genes was conducted using the ABI Q5 Prism sequence detection system (Applied Biosystems, Foster City, CA, United States). The relative mRNA expression levels were calculated by the 2^−ΔΔCT^ method described by Livak and Schmittgen. The primer sequences for each gene are listed in Supplementary Table S1.

### 2.11 Microbial analyses

Colonic digesta samples were sent to Novogene Bioinformatics Technology Co., Ltd. (Beijing, China) for microbiome sequencing analysis. DNA was extracted, and its concentration and purity were measured. A portion of the DNA was diluted to 1 ng/μL and amplified using primers specific to the V_3_–V_4_ regions of the 16S rRNA gene. The PCR products were purified with magnetic beads, quantified, and combined in equal amounts. Library construction was performed using the NEB Next^®^ Ultra™II FS DNA PCR-free Library Prep Kit (New England Biolabs). The library was quantified using Qubit and real-time PCR, and the size distribution was assessed using a bioanalyzer. Sequencing was performed on the Illumina platform based on the effective library concentration and the required data volume. Operational taxonomic units (OTUs) with sequence similarity over 97% were selected for sequence representation. Additionally, the method for detecting SCFA content in colonic digesta was referenced from previous reports (24). The temperature program was as described previously.

### 2.12 Statistical analyses

Data were analyzed using SAS 9.4 software (SAS Inst. Inc., Cary, NC). The normality of the data was checked using the Shapiro–Wilk test. Data from the *in vitro* enzymatic hydrolysis and fermentation experiments were analyzed using *Student’s t*-test. Data from the animal experiment were analyzed using the PROC MIXED procedure with a two-way factorial design. The first factor was the inclusion of MCPC (with or without), and the second factor included three dietary levels: PC, NC1, and NC2. Alpha diversity for the microbiome data was calculated using QIIME 1.7.0. Principal coordinate analysis plots were created based on unweighted unifrac metrics. The relative abundance of microbiota at the phylum and genus levels was log_10_-transformed for statistical analysis. Spearman’s rank correlation analysis was performed to assess correlations between colonic microbiota and SCFAs. Mean values were differentiated using the Tukey method, with a significance level set at 0.05. Results are expressed as the mean ± standard deviation (SD). Differences were considered significant at *p* < 0.05, while 0.05 < *p* < 0.10 was considered a tendency.

## 3 Results

### 3.1 Calcium, phosphorus and reducing sugar content in the supernatant of *in vitro* enzymatic hydrolysis

**Figure 1:**
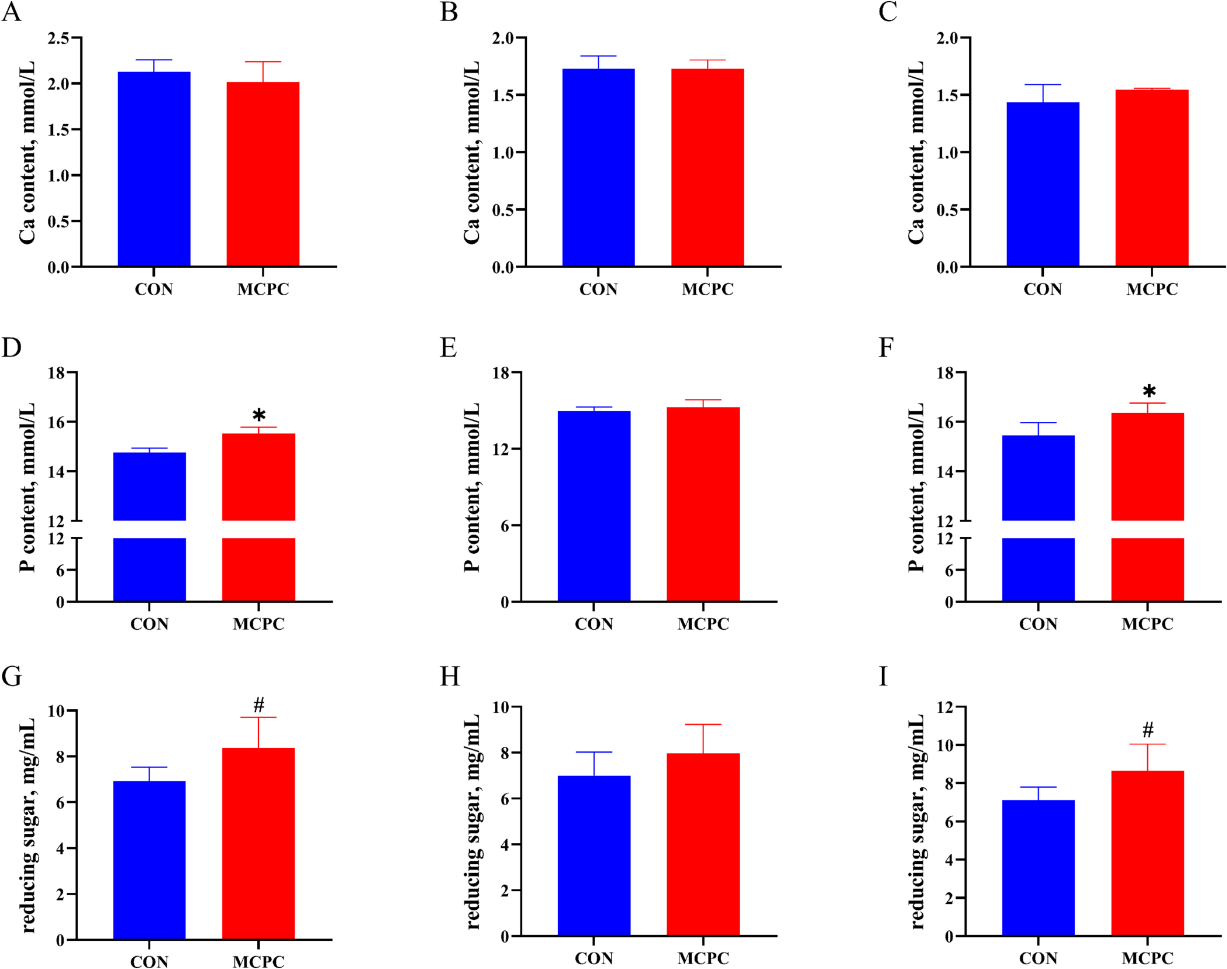
Effects of MCPC on calcium and phosphorus levels in the supernatant of dietary enzymatic hydrolysis. Calcium (Ca) content in the supernatant of enzymatic hydrolysis from PC **(A)**, NC1 **(B)**, and NC2 **(C)** diets; phosphorus (P) content in the supernatant of enzymatic hydrolysis from PC **(D)**, NC1 **(E)**, and NC2 **(F)** diets; reducing sugar content in the supernatant of enzymatic hydrolysis from PC **(G)**, NC1 **(H)**, and NC2 **(I)** diets. MCPC = NSP enzymes and phytase complex. *^, #^ represent *p* < 0.05 and 0.05 < *p* < 0.10. *n* = 4/treatment.

As shown in Figure 1, when compared to the CON treatment, MCPC increased the P (Figures 1D,F, *p* < 0.05) and reducing sugar content (Figures 1G,I, *p* < 0.10) in the supernatant of enzymatic hydrolysis from both PC and NC2 diets. Furthermore, no significant differences in Ca content were observed between the two treatments.

### 3.2 The digestibility of gross energy and crude protein during *in vitro* enzymatic hydrolysis

There were no significant differences in the digestibility of GE and CP between the two treatments during the *in vitro* enzymatic hydrolysis process (Supplementary Table S2, *p* > 0.10).

### 3.3 Accumulated gas production of enzymatic hydrolysis products *in vitro* fermentation

**Figure 2:**
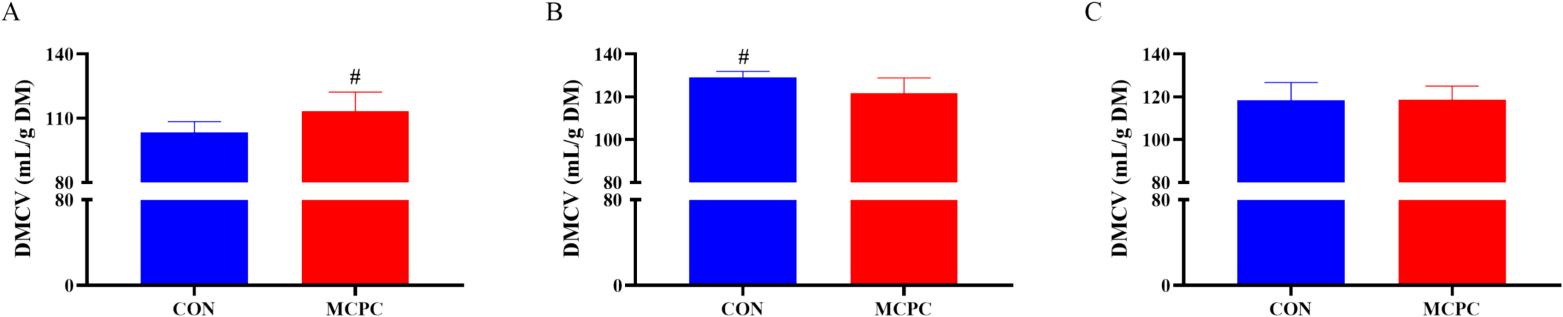
Effects of MCPC on dry matter cumulative volumes of dietary enzymatic hydrolysis products during 48 h of *in vitro* fermentation. **(A)**, dry matter cumulative volumes (DMCV) of the PC enzymatic hydrolysis product; **(B)**, DMCV of the NC1 enzymatic hydrolysis product; **(C)**, DMCV of the NC2 enzymatic hydrolysis product; MCPC = NSP enzymes and phytase complex. ^#^ represents 0.05 < *p* < 0.10. *n* = 4/treatment.

As depicted in Figure 2, MCPC tended to increase (*p* = 0.099) the dry matter cumulative volumes (DMCV) of the PC diet, while it decreased (*p* = 0.099) the DMCV of NC1 enzymatic hydrolysis products after 48 h *in vitro* fermentation, compared to the CON treatment.

### 3.4 Kinetic parameters of dietary enzymatic hydrolysis products *in vitro* fermentation

As presented in Table 2, MCPC significantly enhanced the FRD_0_ value of enzymatic hydrolysis products from both the PC and NC1 diets during *in vitro* fermentation (*p* < 0.05), compared to the CON group. Additionally, MCPC reduced the T_1/2_ of *in vitro* fermentation of enzymatic hydrolysis products from the PC diet (*p* < 0.01), as well as the V_F_ (*p* = 0.082) and K (*p* < 0.05) values for the NC1 diet.

**Table 2:** Effects of MCPC on the kinetic parameters of dietary enzymatic hydrolysis products during *in vitro* fermentation.

Item	CON	MCPC	*p*-value
PC			
V_F_, mL/g DM	102.06 ± 5.35	107.91 ± 6.20	0.263
K	0.16 ± 0.01	0.18 ± 0.02	0.192
FRD_0_^×100^	0.66 ± 0.16	1.12 ± 0.23	0.032
T_1/2_, h	20.31 ± 0.71	15.87 ± 0.51	<0.001
NC1			
V_F_, mL/g DM	125.10 ± 1.34	116.61 ± 6.92	0.082
K	0.21 ± 0.01	0.17 ± 0.01	0.011
FRD_0_^×100^	0.87 ± 0.17	1.28 ± 0.23	0.045
T_1/2_, h	15.27 ± 0.20	15.55 ± 0.31	0.421
NC2			
V_F_, mL/g DM	114.36 ± 5.91	115.96 ± 6.93	0.772
K	0.22 ± 0.02	0.19 ± 0.03	0.218
FRD_0_^×100^	1.08 ± 0.15	1.27 ± 0.15	0.171
T_1/2_, h	14.16 ± 0.22	14.83 ± 1.24	0.392

V_F_, the final asymptotic gas volume; FRD_0_, the fractional rate of degradation at *t*-value = 0 (h^−1^); K, the fractional rate of gas production at particular time (h^−1^); T_1/2_, half-life to asymptote. MCPC = NSP enzymes and phytase complex. *n* = 4/treatment.

### 3.5 Short-chain fatty acids in the supernatant of dietary enzymatic hydrolysis products during *in vitro* fermentation

As shown in Table 3, MCPC significantly increased the acetic acid (AA) level in the supernatant of NC1 enzymatic hydrolysis products during *in vitro* fermentation (*p* < 0.05), compared to the CON group. Additionally, MCPC tended to lower the levels of butyric acid (BA, *p* = 0.057) and propionic acid (PA, *p* = 0.052) in the supernatant of PC enzymatic hydrolysis products during *in vitro* fermentation. No significant differences were found in pH value changes in the supernatant between the two treatments (Supplementary Table S3, *p* > 0.10).

**Table 3:** Effects of MCPC on the concentration of short-chain fatty acids in the supernatant of dietary enzymatic hydrolysis products during *in vitro* fermentation.

Item	CON	MCPC	*p*-value
PC			
AA	24.49 ± 0.24	22.66 ± 1.74	0.122
BA	3.51 ± 0.09	3.07 ± 0.31	0.057
PA	4.76 ± 0.07	4.33 ± 0.30	0.052
VA	0.66 ± 0.02	0.60 ± 0.06	0.171
IBA	0.46 ± 0.02	0.43 ± 0.05	0.320
IVA	1.14 ± 0.06	1.11 ± 0.07	0.675
NC1			
AA	22.20 ± 0.14	22.88 ± 0.33	0.027
BA	4.11 ± 0.15	3.82 ± 0.39	0.270
PA	5.93 ± 0.04	5.70 ± 0.50	0.451
VA	0.83 ± 0.01	0.81 ± 0.07	0.585
IBA	0.56 ± 0.02	0.53 ± 0.05	0.320
IVA	1.47 ± 0.06	1.48 ± 0.11	0.868
NC2			
AA	22.42 ± 0.93	21.31 ± 0.76	0.160
BA	3.71 ± 0.25	3.56 ± 0.26	0.507
PA	5.41 ± 0.20	5.01 ± 0.29	0.102
VA	0.86 ± 0.02	0.84 ± 0.05	0.730
IBA	0.56 ± 0.02	0.54 ± 0.03	0.356
IVA	1.50 ± 0.10	1.47 ± 0.08	0.645

AA, acetic acid; BA, butyric acid; PA, propionic acid; VA, valeric acid; IBA, isobutyric acid; IPA, isovaleric acid. MCPC = NSP enzymes and phytase complex. *n* = 4/treatment.

### 3.6 Expression of nutrient transport gene in the duodenum of growing pigs

**Figure 3:**
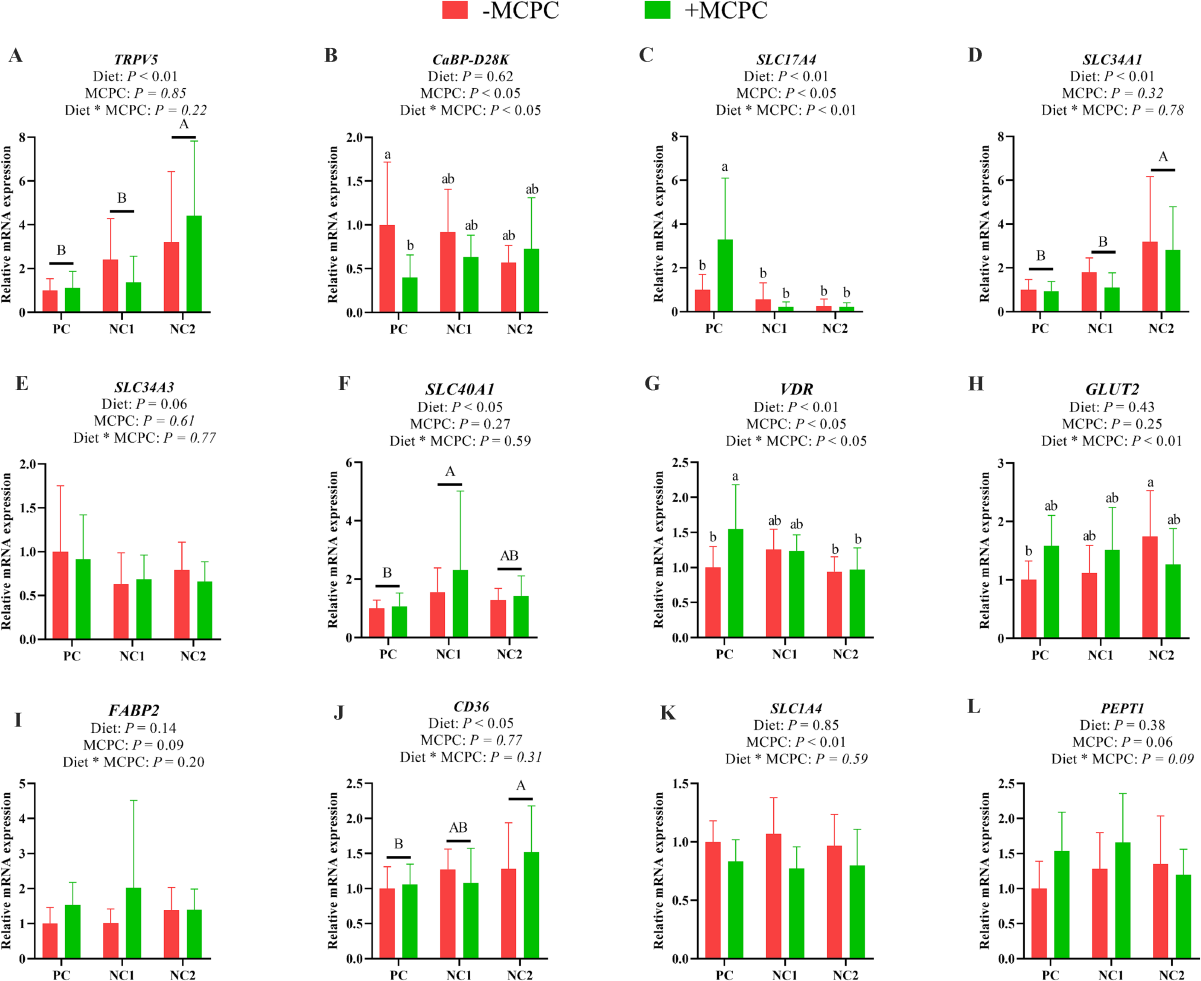
Effects of supplemental MCPC in diets with varying nutritional levels on the relative expression of nutrient transport genes in the duodenum of growing pigs. **(A)**, *TRPV5*; **(B)**, *CaBP-D28K*; **(C)**, *SLC17A4*; **(D)**, *SLC34A1*; **(E)**, *SLC34A3*; **(F)**, *SLC40A1*; **(G)**, *VDR*; **(H)**, *GLUT2*; **(I)**, *FABP2*; **(J)**, *CD36*; **(K)**, *SLC1A4*; **(L)**, *PEPT1*; MCPC = NSP enzymes and phytase complex; PC = positive control diet; NC1 = negative control 1 diet; NC2 = negative control 2 diet. A, B and a, b, c indicate significant differences at *p* < 0.05. *n* = 12/treatment.

As presented in Figure 3, the relative expression levels of *TRPV5*, *SLC34A1*, and *CD36* genes in the duodenum of pigs fed the NC2 diet were significantly higher than those in pigs fed the PC and NC1 diets. Additionally, the relative expression of the *SLC40A1* gene was significantly greater in pigs fed the NC1 diet compared to those on the PC diet (*p* < 0.05). The inclusion of MCPC in the diet significantly increased the expression levels of the *SLC17A4* and *VDR* genes in the duodenum (*p* < 0.05), while reducing the expression levels of *CaBP-D28K* and *SLC1A4* (*p* < 0.05). The interaction between diet and MCPC significantly influenced the relative expression levels of the *CaBP-D28K*, *SLC17A4*, *VDR*, and *GLUT2* genes in the duodenum of growing pigs (*p* < 0.05).

### 3.7 Expression of nutrient transport gene in the jejunum of growing pigs

**Figure 4:**
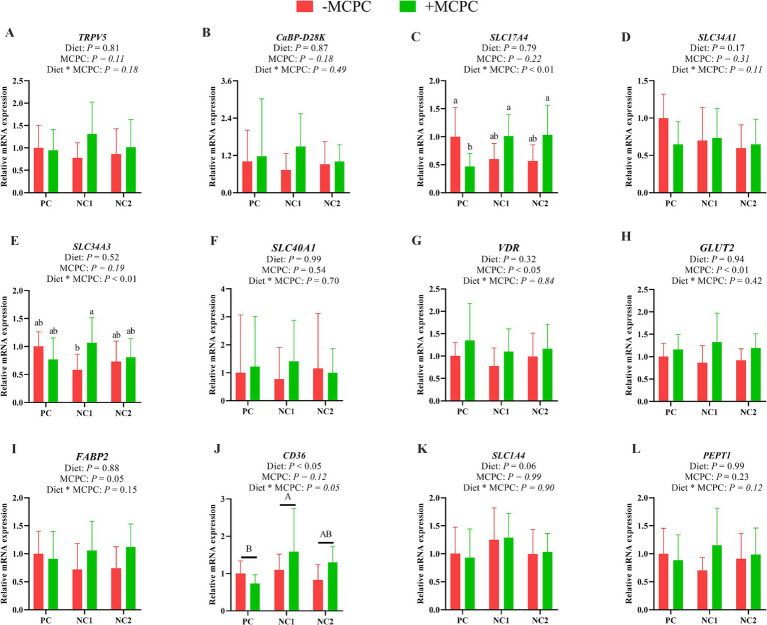
Effects of supplemental MCPC in diets with varying nutritional levels on the relative expression of nutrient transport genes in the jejunum of growing pigs. **(A)**, *TRPV5*; **(B)**, *CaBP-D28K*; **(C)**, *SLC17A4*; **(D)**, *SLC34A1*; **(E)**, *SLC34A3*; **(F)**, *SLC40A1*; **(G)**, *VDR*; **(H)**, *GLUT2*; **(I)**, *FABP2*; **(J)**, *CD36*; **(K)**, *SLC1A4*; **(L)**, *PEPT1*; MCPC = NSP enzymes and phytase complex; PC = positive control diet; NC1 = negative control 1 diet; NC2 = negative control 2 diet. A, B and a, b, c indicate significant differences at *p* < 0.05. *n* = 12/treatment.

As shown in Figure 4, the relative expression of the *CD36* gene in the jejunum of pigs fed the NC1 diet was significantly higher than that of the PC diet group (*p* < 0.05). The addition of MCPC to the diet significantly upregulated the relative expression levels of the *VDR*, *GLUT2* and *FABP2* genes in the jejunum. The interaction effect of diet and MCPC significantly affected the relative expression levels of the *SLC17A4* and *SLC34A3* genes in the jejunum (*p* < 0.05). The relative expression of the *SLC17A4* gene in the jejunum of growing pigs fed the PC, NC1 + MCPC, and NC2 + MCPC diets was significantly higher compared to the PC + MCPC group. In the NC1 diet, the relative expression of the *SLC34A3* gene in the jejunum of pigs in the NC1 + MCPC treatment was significantly increased compared to the NC1 treatment (*p* < 0.05).

### 3.8 Colonic microbial diversity of growing-finishing pigs

**Figure 5:**
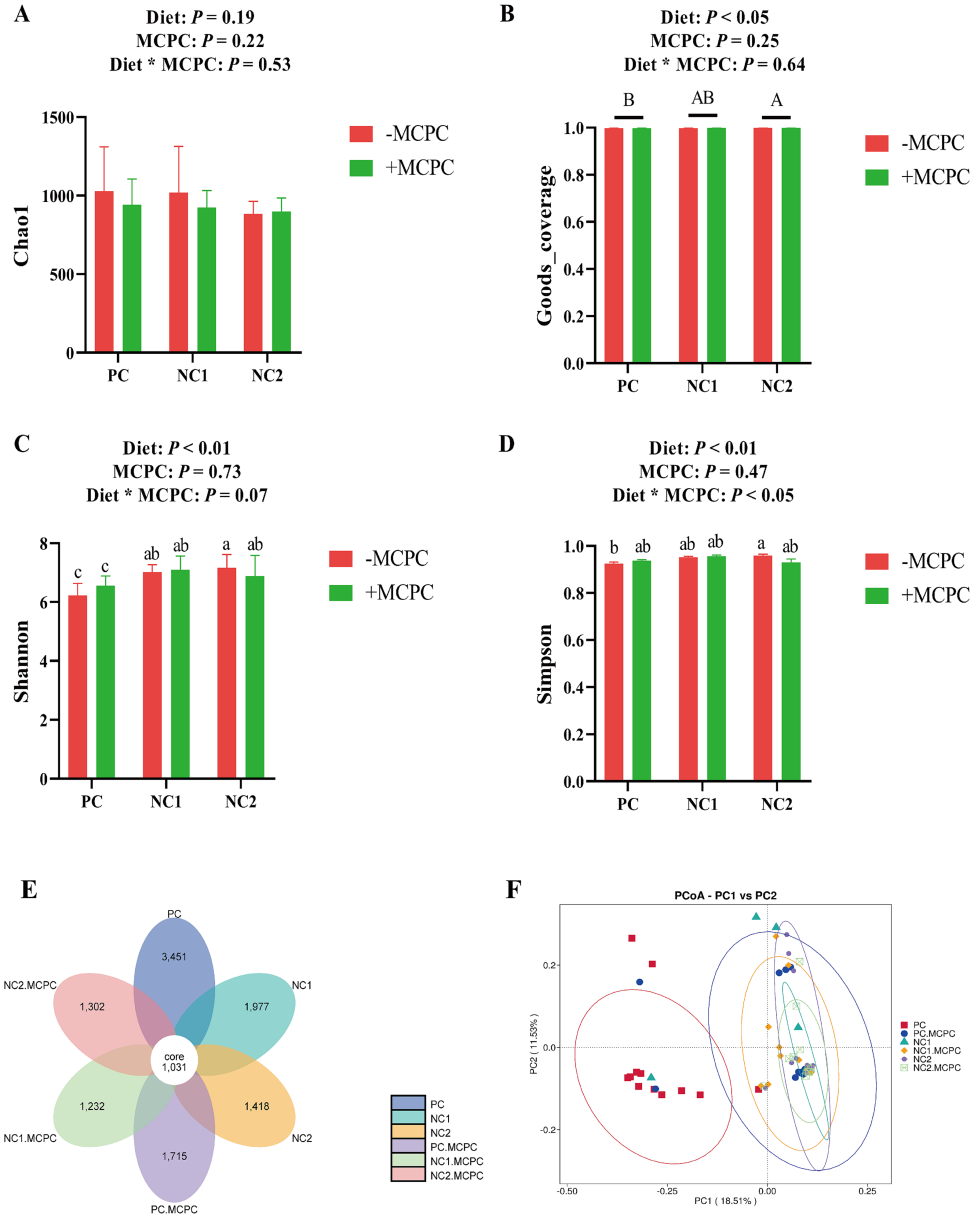
Effects of supplemental MCPC in diets with varying nutritional levels on cecal microbial diversity in growing-finishing pigs. **(A–D)** Alpha diversity index; **(E)** OUT flower petal diagram; **(F)** PCoA (unweighted Unifrac). MCPC = NSP enzymes and phytase complex. A, B and a, b, c indicate significant differences at *p* < 0.05. *n* = 12/treatment.

As presented in Figure 5, the Shannon and Goods-coverage indices of colonic microbiota in growing pigs fed the NC2 diet were significantly higher compared to those fed the PC diet (*p* < 0.05). However, the inclusion of MCPC in the diet did no significant alter the Chao1, Goods-coverage, Shannon, and Simpson indices of colonic microbiota in growing pigs. The interaction between diet and MCPC significantly affected the Shannon (*p* = 0.07) and Simpson (*p* < 0.05) indices. The PC, PC + MCPC, NC1, NC1 + MCPC, NC2, and NC2 + MCPC treatments had 3,451, 1,715, 1,977, 1,232, 1,418, and 1,306 unique feature sequences, respectively, along with 1,031 shared feature sequences (Figure 4E). Moreover, in the principal coordinate analysis (PCoA, unweighted unifrac) showed that samples positioned closer together had similar microbial structures, while those farther apart exhibited more dissimilarity. The PCoA indicated that the PC treatment was distinct from the others, indicating significant differences in community structure, while the other treatments (excluding PC) showed similar distributions of colonic fecal bacterial communities (Figure 4F).

### 3.9 Relative abundance of colon chyme bacteria at phylum level in growing pigs

Table 4 presents the top 10 colonic microbial compositions at the phylum level for each treatment group. Compared to the NC1 and NC2 diets, the relative abundances of *Firmicutes*, *Proteobacteria*, *Actinobacteriota*, and *Verrucomicrobiota* were significantly higher in the colons of pigs fed the PC diet (*p* < 0.05), while the relative abundances of *Bacteroidota* and *Spirochaetota* were significantly lower (*p* < 0.05). Furthermore, the inclusion of MCPC in the diet significantly increased the relative abundance of *Bacteroidota* and decreased that of *Verrucomicrobiota* in the colons of growing pigs (*p* < 0.05). There were also trends toward increased *Spirochaetota* (*p* = 0.06) and decreased *Actinobacteriota* (*p* = 0.07) and *Euryarchaeota* (*p* = 0.09) relative abundances. Additionally, the interaction between diet and MCPC significantly influenced the relative abundances of *Bacteroidota*, *Proteobacteria*, *Spirochaetota*, *Verrucomicrobiota*, and *Campilobacterota* (*p* < 0.05).

**Table 4:** Effects of supplemental MCPC in diets with varying nutritional levels on the relative abundance of colon chyme bacteria at the phylum level in growing pigs.

Item	PC		NC1		NC2		Basal diet			MCPC		*p*-value		
	−MCPC	+MCPC	−MCPC	+MCPC	−MCPC	+MCPC	PC	NC1	NC2	−MCPC	+ MCPC	Diet	MCPC	Diet × MCPC
*Firmicutes*	86.18 ± 3.60	83.61 ± 3.52	78.01 ± 5.23	78.43 ± 5.30	73.34 ± 7.81	74.38 ± 8.67	84.90 ± 3.79^a^	78.22 ± 5.27^b^	73.86 ± 8.27^c^	79.18 ± 7.87	78.81 ± 7.27	<0.001	0.835	0.637
*Bacteroidota*	4.03 ± 2.03^c^	10.71 ± 3.65^b^	17.15 ± 6.49^ab^	16.79 ± 6.26^ab^	21.73 ± 7.04^a^	20.90 ± 8.39^a^	7.37 ± 4.46^b^	16.97 ± 6.38^a^	21.32 ± 7.76^a^	14.31 ± 9.39	16.13 ± 7.65	<0.001	0.005	0.002
*Proteobacteria*	3.46 ± 2.22^a^	1.83 ± 1.80^b^	1.24 ± 0.67^b^	1.91 ± 1.99^b^	1.41 ± 0.48^b^	1.31 ± 0.51^b^	2.64 ± 2.18^a^	1.57 ± 1.52^b^	1.36 ± 0.50^b^	2.04 ± 1.70	1.68 ± 1.60	0.007	0.111	0.024
*Actinobacteriota*	2.36 ± 1.50	1.84 ± 1.59	1.13 ± 0.96	0.80 ± 0.42	1.08 ± 0.53	1.00 ± 0.90	2.10 ± 1.57^a^	0.96 ± 0.76^b^	1.04 ± 0.74^b^	1.52 ± 1.23	1.21 ± 1.17	<0.001	0.068	0.870
*Spirochaetota*	0.34 ± 0.34	0.78 ± 0.46	1.36 ± 1.63	1.41 ± 1.48	1.46 ± 1.87	1.61 ± 1.71	0.56 ± 0.46^b^	1.40 ± 1.56^a^	1.59 ± 1.79^a^	1.09 ± 1.54	1.28 ± 1.38	0.001	0.058	0.050
*Euryarchaeota*	1.12 ± 1.31	0.42 ± 0.38	0.39 ± 0.48	0.27 ± 0.43	0.50 ± 0.54	0.42 ± 0.64	0.77 ± 1.02	0.33 ± 0.46	0.46 ± 0.60	0.67 ± 0.92	0.37 ± 0.50	0.282	0.085	0.886
*Verrucomicrobiota*	0.39 ± 0.21^a^	0.08 ± 0.07^b^	0.02 ± 0.04^c^	0.01 ± 0.01^c^	0.01 ± 0.01^c^	0.02 ± 0.03^bc^	0.23 ± 0.22^a^	0.02 ± 0.03^b^	0.02 ± 0.02^b^	0.14 ± 0.22	0.04 ± 0.06	<0.001	0.045	0.009
*Desulfobacterota*	0.19 ± 0.07	0.15 ± 0.07	0.19 ± 0.06	0.21 ± 0.15	0.23 ± 0.11	0.21 ± 0.06	0.17 ± 0.07	0.20 ± 0.12	0.22 ± 0.09	0.21 ± 0.09	0.19 ± 0.11	0.309	0.197	0.560
*Campilobacterota*	0.08 ± 0.13^a^	0.02 ± 0.01^c^	0.02 ± 0.01^bc^	0.07 ± 0.07^ab^	0.06 ± 0.06^abc^	0.04 ± 0.03^abc^	0.05 ± 0.09	0.05 ± 0.05	0.05 ± 0.05	0.06 ± 0.08	0.04 ± 0.05	0.500	0.489	0.025
*Patescibacteria*	0.02 ± 0.02	0.02 ± 0.02	0.02 ± 0.01	0.02 ± 0.02	0.02 ± 0.01	0.05 ± 0.07	0.02 ± 0.02	0.02 ± 0.02	0.03 ± 0.05	0.02 ± 0.02	0.03 ± 0.04	0.424	0.362	0.361

MCPC, NSP enzymes and phytase complex. ^a, b, c^ Means with no common letters differ at *p* < 0.05. *n* = 12/treatment.

### 3.10 Relative abundance of colon chyme bacteria at genus level in growing pigs

Table 5 shows the top 15 microbial compositions at the genus level. Compared to the PC diet, pigs fed the NC1 and NC2 diets had significantly higher relative abundances of *Streptococcus*, *Megasphaera*, *Agathobacter*, *Treponema*, *Alloprevotella*, and *Phascolarctobacterium* (*p* < 0.05), while the relative abundances of *Lactobacillus*, *Clostridium_sensu_stricto_1*, *Terrisporobacter*, *Escherichia-Shigella*, *Corynebacterium*, and *Turicibacter* were significantly lower (*p* < 0.05). The addition of MCPC to the diet led to a significant increase in the relative abundance of *Prevotella* and *Phascolarctobacterium* in the colons of pigs (*p* < 0.05), while the relative abundance of *Clostridium_sensu_stricto_1* was significantly decreased (*p* < 0.05). There was also a trend toward increased *Streptococcus* (*p* = 0.06). The interaction between diet and MCPC significantly influenced the relative abundances of *Megasphaera*, *Prevotella*, *Escherichia-Shigella*, *Alloprevotella*, *Mitsuokella*, and *Turicibacter* (*p* < 0.05).

**Table 5:** Effects of supplemental MCPC in diets with varying nutritional levels on the relative abundance of colon chyme bacteria at the genus level in growing pigs.

Item	PC		NC1		NC2		Basal diet			MCPC		*p*-value		
	−MCPC	+MCPC	−MCPC	+MCPC	−MCPC	+MCPC	PC	NC1	NC2	−MCPC	+ MCPC	Diet	MCPC	Diet × MCPC
*Lactobacillus*	13.65 ± 9.99	10.08 ± 8.22	4.74 ± 5.16	6.95 ± 8.47	11.56 ± 10.89	6.82 ± 3.77	11.86 ± 9.32^a^	5.84 ± 7.10^b^	9.19 ± 8.49^a^	9.98 ± 9.80	7.95 ± 7.31	0.001	0.609	0.241
*Streptococcus*	10.12 ± 9.05	11.80 ± 6.26	16.81 ± 6.01	17.34 ± 6.98	14.15 ± 7.19	24.17 ± 11.88	10.96 ± 7.83^b^	17.07 ± 6.52^a^	19.16 ± 11.03^a^	13.70 ± 8.01	17.77 ± 10.10	0.002	0.057	0.363
*Clostridium_sensu_stricto_1*	24.70 ± 9.24	21.98 ± 8.45	15.18 ± 5.04	9.75 ± 5.81	7.61 ± 4.35	5.57 ± 3.96	23.34 ± 8.96^a^	12.47 ± 6.08^b^	6.59 ± 4.28^c^	15.83 ± 9.60	12.44 ± 9.42	<0.001	0.011	0.336
*Terrisporobacter*	7.17 ± 3.61	6.71 ± 2.74	6.00 ± 2.74	3.79 ± 2.15	3.85 ± 2.60	3.36 ± 2.32	6.94 ± 3.21^a^	4.90 ± 2.70^b^	3.61 ± 2.48^b^	5.67 ± 3.32	4.62 ± 2.83	<0.001	0.058	0.329
*Megasphaera*	0.29 ± 0.33^b^	1.69 ± 1.72^a^	2.63 ± 1.70^a^	4.22 ± 5.08^a^	3.48 ± 3.82^a^	1.80 ± 1.20^a^	0.99 ± 1.42^b^	3.42 ± 3.87^a^	2.64 ± 2.95^a^	2.13 ± 2.77	2.57 ± 3.38	<0.001	0.218	0.028
*Agathobacter*	1.44 ± 0.90	1.69 ± 0.68	2.25 ± 0.89	2.74 ± 1.36	2.47 ± 0.93	2.43 ± 1.78	1.56 ± 0.81^b^	2.49 ± 1.18^a^	2.45 ± 1.42^a^	2.05 ± 1.01	2.28 ± 1.42	0.004	0.440	0.387
*Prevotella*	0.15 ± 0.21^b^	1.06 ± 0.76^a^	3.32 ± 2.56^a^	2.84 ± 1.86^a^	3.73 ± 2.78^a^	3.49 ± 1.82^a^	0.63 ± 0.73^b^	3.08 ± 2.25^a^	3.61 ± 2.35^a^	2.40 ± 2.72	2.46 ± 1.87	<0.001	0.033	0.015
*Escherichia-Shigella*	1.50 ± 2.06^a^	0.75 ± 0.93^ab^	0.39 ± 0.25^ab^	1.18 ± 1.78^a^	0.44 ± 0.36^ab^	0.19 ± 0.10^b^	1.13 ± 1.64^a^	0.79 ± 1.33^a^	0.32 ± 0.29^b^	0.78 ± 1.32	0.71 ± 1.23	0.002	0.308	0.038
*Treponema*	0.33 ± 0.33	0.71 ± 0.47	1.21 ± 1.67	1.27 ± 1.43	1.40 ± 1.82	1.45 ± 1.59	0.52 ± 1.45^b^	1.24 ± 1.56^a^	1.42 ± 1.71^a^	0.98 ± 1.51	1.14 ± 1.30	0.004	0.069	0.131
*Alloprevotella*	0.15 ± 0.18^b^	0.66 ± 0.38^a^	1.44 ± 0.96^a^	1.31 ± 0.83^a^	2.27 ± 1.47^a^	1.70 ± 1.38^a^	0.41 ± 0.39^b^	1.38 ± 0.90^a^	1.99 ± 1.45^a^	1.29 ± 1.34	1.23 ± 1.05	<0.001	0.081	<0.001
*Corynebacterium*	0.53 ± 0.58	0.74 ± 1.46	0.13 ± 0.10	0.20 ± 0.25	0.19 ± 0.17	0.13 ± 0.15	0.63 ± 1.12^a^	0.16 ± 0.19^b^	0.16 ± 0.16^b^	0.28 ± 0.40	0.36 ± 0.90	0.002	0.485	0.450
*Phascolarctobacterium*	0.23 ± 0.17	0.26 ± 0.12	0.16 ± 0.08	0.45 ± 0.28	0.55 ± 0.20	0.97 ± 1.34	0.24 ± 0.15^b^	0.31 ± 0.25^b^	0.76 ± 0.98^a^	0.31 ± 0.23	0.56 ± 0.85	<0.001	0.021	0.257
*Mitsuokella*	0.07 ± 0.09^b^	0.67 ± 0.83^a^	0.72 ± 1.23^a^	0.52 ± 0.42^a^	0.59 ± 0.71^a^	0.36 ± 0.26^a^	0.37 ± 0.66	0.62 ± 0.93	0.47 ± 0.55	0.46 ± 0.87	0.52 ± 0.57	0.606	0.497	0.024
*Bifidobacterium*	0.47 ± 1.12	0.55 ± 0.77	0.43 ± 0.71	0.25 ± 0.20	0.52 ± 0.38	0.47 ± 0.61	0.51 ± 0.96	0.34 ± 0.53	0.49 ± 0.51	0.47 ± 0.80	0.43 ± 0.59	0.178	0.877	0.166
*Turicibacter*	1.60 ± 0.83^a^	1.30 ± 0.64^a^	1.02 ± 0.40^ab^	0.67 ± 0.46^bc^	0.33 ± 0.14^c^	0.54 ± 0.39^c^	1.45 ± 0.76^a^	0.85 ± 0.47^b^	0.44 ± 0.31^c^	0.98 ± 0.75	0.84 ± 0.61	<0.001	0.364	0.045

MCPC = NSP enzymes and phytase complex. ^a, b, c^ Means with no common letters differ at *p* < 0.05. *n* = 12/treatment.

### 3.11 Short-chain fatty acids in colonic chyme of growing pigs

**Figure 6:**
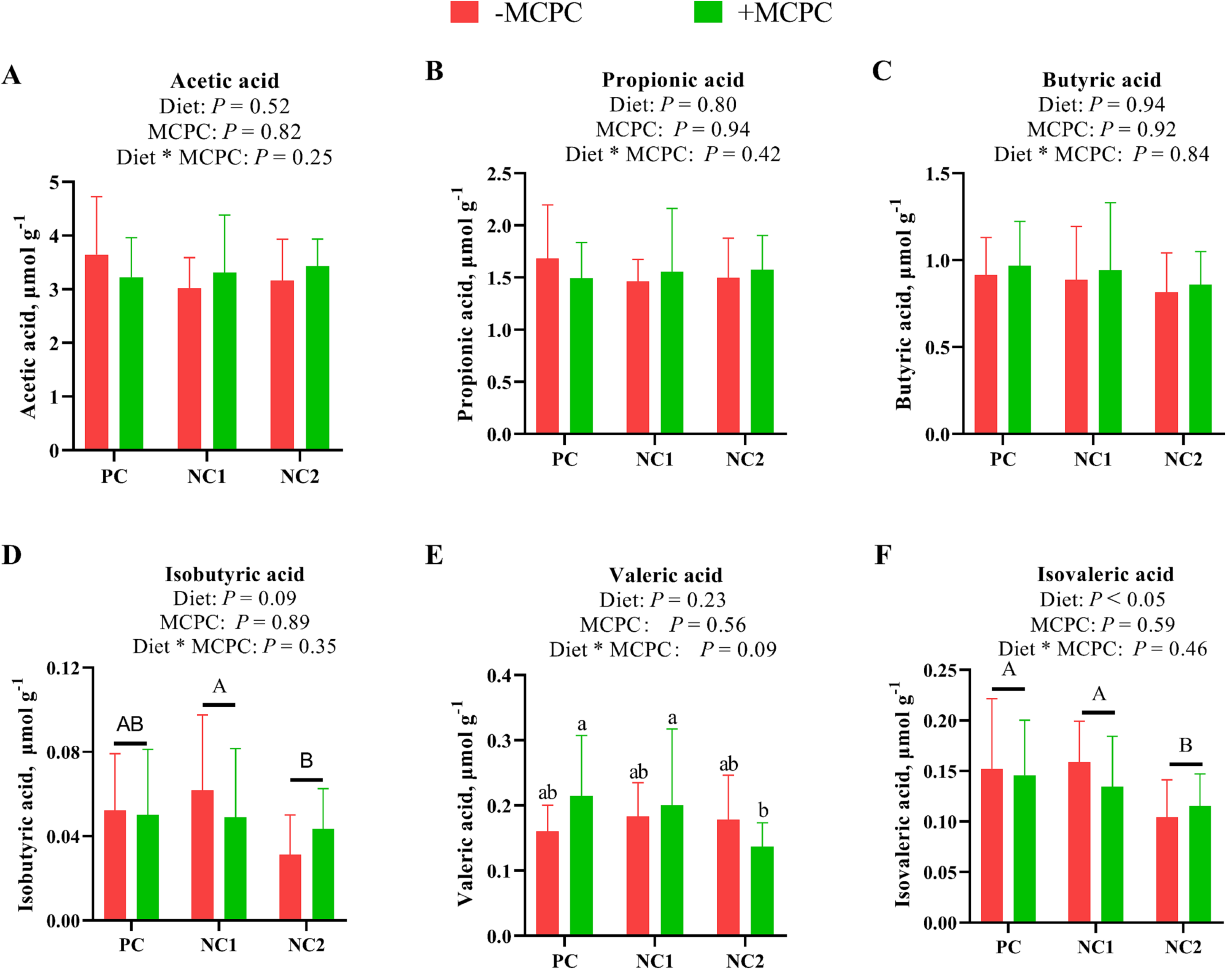
Effects of supplemental MCPC in diets with different nutrition levels on content of short-chain fatty acids in the colonic chyme of growing-finishing pigs. **(A)**, Acetic acid; **(B)**, Propionic acid; **(C)**, Butyric acid; **(D)**, Isobutyric acid; **(E)**, Valeric acid; **(F)**, Isovaleric acid. MCPC = NSP enzymes and phytase complex. A, B and a, b, c indicate significant differences at *p* < 0.05. *n* = 12/treatment.

As shown in Figure 6, the isovaleric acid (IVA) content in the colonic digesta of growing pigs fed the NC2 diet was significantly lower compared to those fed the PC and NC1 diets (*p* < 0.05). Moreover, isobutyric acid (IBA) levels in the colonic digesta of pigs fed the NC2 diet were 27% lower than in those fed the NC1 diet (*p* < 0.05). The addition of MCPC to the diet had no significant impact on the levels of various SCFAs in the colonic digesta (*p* > 0.10). The interaction between diet and MCPC had a tendency to influence the PA content (*p* = 0.09). Furthermore, the PA content in the colonic digesta of pigs on the NC2 + MCPC treatment was significantly lower compared to the PC + MCPC and NC1 + MCPC treatments.

### 3.12 Correlation between colonic microbiota and short-chain fatty acids in growing pigs

**Figure 7:**
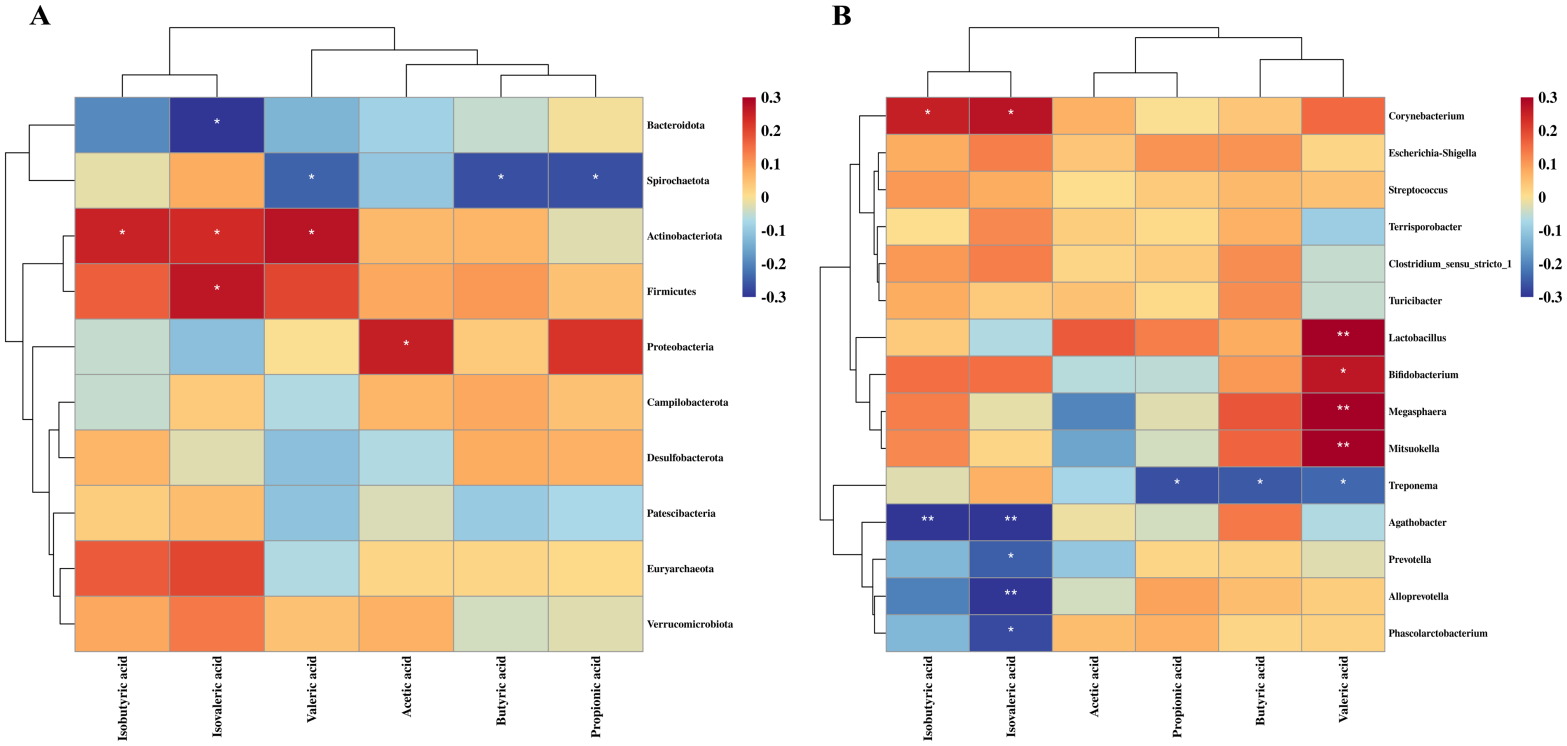
Relationship between colonic microbiota and short-chain fatty acids in growing pigs. **(A)** Correlation between microbial communities at the phylum level and short-chain fatty acids. **(B)** Correlation between microbial communities at the genus level and short-chain fatty acids. *, *p* < 0.05; **, *p* < 0.01.

As shown in Figure 7, Spearman’s rank correlation analysis was performed to investigate the relationship between colonic bacterial abundance and SCFA concentrations. Several microbiota displayed significant positive or negative correlations with SCFA levels. *Bacteroidota*, *Agathobacter*, *Prevotella*, *Alloprevotella* and *Phascolarctobacterium* were negatively correlated with IVA (*p* < 0.05). *Spirochaetota* and *Treponema* showed a significant negative correlation with PA, BA and valeric acid (VA) (*p* < 0.05). In contrast, *Actinobacteria*, *Corynebacterium* and *Firmicutes* were positively correlated with IVA, IBA, and/or VA (*p* < 0.05). *Lactobacillus*, *Bifidobacterium*, *Mitsuokella* and *Megasphaera* were significantly positively correlated with VA (*p* < 0.05).

## 4 Discussion

There is a growing interest in reducing costs, optimizing feed formulations and improving production performance by applying exogenous multi-enzymes to mitigate dietary anti-nutrients such as phytic acid and non-starch polysaccharides. However, limited studies have been conducted on how multi-enzyme supplementation enhances foregut nutrient transport and posterior gut microbial fermentation, indicating the need for further exploration. In the present study, *in vitro* simulated digestion was initially used to evaluate the specific processes of digestion in the foregut and fermentation in the hindgut. Phytase breaks down phytate in feed materials into inorganic phosphorus and myo-inositol, while also releasing nutrients, including starch and proteins, that were previously chelated by phytate (25). Xylanase and *β*-glucanase reduce intestinal viscosity, promoting the release of small oligosaccharides, which are then further hydrolyzed into reducing sugars (26). In this study, MCPC improved the P and reducing sugar levels in the supernatant from enzymatic hydrolysis of diets, corroborating these findings. Previous studies have demonstrated that phytase enhances the rapid breakdown of phosphorus and the release of other nutrients during *in vitro* digestion (27, 28). Additionally, xylanase and β-glucanase degrade dietary xylan and β-glucan, improving feed energy bioavailability by releasing reducing sugars such as glucose (29, 30). However, no significant changes in the Ca content of the supernatant or in the digestibility of GE and CP in the enzymatic hydrolysis products were observed. The *in vitro* digestibility of GE is typically lower than *in vivo* physiological conditions (31). Previous studies indicated that during NSP enzymatic hydrolysis, intermediate products like oligosaccharides or partially degraded fibers may form, which can bind to digestive enzymes (e.g., pancreatic enzymes) and inhibit proteins and starch degradation (32). These intermediate products might also release minerals bound to phytate, facilitating their recombination with proteins to form indigestible complexes (33, 34). Furthermore, excessive or insufficient enzyme activity can lead to over-degradation of nutrients, resulting in the accumulation of intermediate products that are hard to absorb, potentially disturbing nutrient balance and endogenous enzyme secretion (35, 36). This study, therefore, indicates that the presence of specific intermediate products during NSP enzymatic hydrolysis may hinder the digestion and absorption of Ca, GE and CP, without affecting the effective release of P and reducing sugars. This emphasizes the importance of optimizing enzyme activity for better nutrient utilization in animal diets.

The intestinal microbiome plays a vital role in enhancing nutrient digestion and absorption, supporting gut health, immune function, and metabolic processes (37, 38). Microbial fermentation generates gases that provide hydrogen for further microbial metabolism (39). By measuring gas production at various time points, the dynamics of the fermentation process can be estimated, serving as an indicator of microbial activity (40, 41). In our study, MCPC increased the cumulative gas production from the fermentation of PC feed over a 48 h period. Several factors, including anti-nutritional elements, nutrient polymerization, and substrate availability, have been found to influence microbial fermentation (42, 43). The fermentation rate at the start (FRD_0_) reflects the availability of the substrate or the degree of its breakdown at the beginning of fermentation. The time needed for the substrate to degrade to half of its original concentration during fermentation is termed T_1/2_ (20). MCPC significantly raised the FRD_0_ of enzymatic products from PC and NC1 diets, while reducing the T_1/2_ of PC enzymatic products. This suggests that the substrate for MCPC hydrolysis likely has a higher proportion of monosaccharides or smaller molecules that are readily fermentable early in the fermentation process, which promotes rapid fermentation and enhances the initial microbial fermentation rate. The lower T_1/2_ value may indicate that fermentation mainly occurs in the proximal and distal parts of the hindgut with the action of MCPC, rather than in the distal colon (44). Furthermore, during the fermentation of NC1 enzymatic products, the V_F_ and K values were lower in the MCPC group, despite the higher FRD_0_. Since the fermentation substrates in both treatments originate from fully enzymatically hydrolyzed feed with the same additive dosage, it is logical that when the nutrient content available for fermentation is significantly reduced in the MCPC group, both the K value and V_F_ would decrease. However, despite the lower total nutrient levels, the increased proportion of oligosaccharides, monosaccharides, and soluble fibers (as indicated by the increase in FRD_0_) may improve fermentation efficiency.

Alterations in gut microbiota can be evaluated by measuring the production of SCFAs. Enzymes can enhance microbial composition by degrading substrates and increasing SCFA levels, which are subsequently utilized by the body (45). SCFAs also reduce intestinal pH value, inhibiting the growth of pathogenic bacteria and supporting the proliferation of beneficial microbiota (46). It has been demonstrated that SCFAs are affected not only by microbial composition changes but also by dietary types (47). The rise in AA levels in the supernatant of NC1 enzymatic products corroborates the theory that MCPC efficiently breaks down complex fibers, thus providing more fermentable substrates for microbial fermentation. Additionally, this might also indicate that MCPC promotes alterations in the gut microbiota, such as increases in *Prevotella*, *Streptococcus,* and *Bacteroidota* (48). The reduction in BA and PA during the fermentation of PC enzymatic hydrolysis products could be due to SCFAs produced during the initial rapid fermentation phase. These SCFAs are presumably consumed by microbes (49) or converted into other compounds (50). In summary, MCPC potentially enhances the fermentation efficiency and degradation rate of substrates in the hindgut by effectively hydrolyzing various complex substrates and enhancing the microbiota composition.

Based on these findings, animal experiments were conducted to explore whether MCPC further improves nutrient absorption in the intestine following effective enzymatic hydrolysis of the diet, as well as to confirm its impact on hindgut fermentation and microbial composition. Our earlier study, using the same growing pigs, examined growth performance and bone mineralization and showed that dietary MCPC supplementation significantly improved the AID of Ca (+33.2%) and P (+115.7%), with no significant impact on the AID of GE and CP. However, MCPC supplementation significantly enhanced the apparent total tract digestibility (ATTD) of GE, CP, P and acid detergent fiber (ADF) in growing pigs (31). In this study, we found that the effects of MCPC on *in vitro* enzymatic hydrolysis in the foregut were consistent with the *in vivo* AID results, particularly in terms of the effective release of P and the lack of significant changes in GE and CP digestibility. After confirming the enzymatic hydrolysis effects of MCPC on substrates, we explored its influence on the expression of intestinal nutrient transport genes. The current study revealed that MCPC upregulated the relative expression of intestinal *SLC17A4*, *VDR*, *GLUT2* and *FABP2* genes, while downregulating the expression of *CaBP-D28K* and *SCL1A4* genes in growing pigs. This suggests that MCPC plays a beneficial role in enhancing the absorption of nutrients such as minerals, fatty acids, and carbohydrates in the intestine. High P intake upregulates the expression of *SLC17A4* via a feedback mechanism of intestinal transporters, promoting the excretion and metabolism of excess phosphorus (51). The upregulation of *VDR* facilitates vitamin D metabolism and enhances the Ca and P absorption (52). Glucose absorption is mediated by the *GLUT2* transporter (53), and its expression increases with improved substrate utilization (54). *FABP2* primarily supports the uptake of fatty acids in the intestine (55). As previously noted, MCPC increased both P and reducing sugar concentrations in the supernatant of enzymatic hydrolysis products. This further suggests that MCPC, by increasing the expression of intestinal transporters such as *SLC17A4*, *GLUT2*, and *VDR*, may work synergistically to improve the absorption of both phosphorus and glucose. This dual enhancement of digestion and absorption likely indicates a coordinated improvement in nutrient utilization mediated by these transporters. Additionally, MCPC downregulated the expression of *CaBP-D28K* in the duodenum, likely due to its effect on enhancing Ca digestion and absorption in growing pigs. This increase in luminal Ca concentration leads to passive diffusion, reducing the reliance on active transport proteins (56–58). Similarly, *TRPV5* plays a key role in maintaining Ca homeostasis. In this study, the expression of *TRPV5* in the duodenum increased under reduced dietary nutrient levels (NC2), likely as an adaptive response to meet the increased demand for Ca absorption in low Ca and P conditions. A previous study has shown that increased dietary calcium levels or microbial phytase supplementation reduces the expression of Ca transporters in the jejunum or colon (56). Additionally, reduced dietary Ca and P levels have been linked to increased expression of *SLC34A1* and *SLC34A3* in the jejunum (59). In our study, the expression of *SLC17A4*, *SLC34A3,* and *SLC34A1* genes in the duodenum varied to different extents with reduced dietary nutrient levels. However, no significant differences were found in the gene expression of *SLC17A4* and *SLC34A3* in the duodenum, as well as *SLC17A4*, *SLC34A1,* and *SLC34A3* in the jejunum between the PC group and the NC1 + MCPC and NC2 + MCPC groups, suggesting consistent changes. These findings indicate that the addition of MCPC to high-fiber diets may meet the Ca and P utilization requirements at normal nutritional levels. Overall, considering the improvements in nutrient digestion and the expression of intestinal nutrient transport genes, MCPC demonstrates significant potential to enhance the digestion and absorption of nutrients, contributing to better growth performance and greater farming efficiency in pigs.

Our previous study demonstrated the significant role of hindgut microbial fermentation in overall intestinal digestion, especially when considering the differences in AID and ATTD in growing pigs (31), which further supports the current findings. The results of this study showed that MCPC supplementation increased the abundance of *Bacteroidota*, *Prevotella*, *Phascolarctobacterium* and other microbes, providing evidence for the microbial changes seen in SCFAs during *in vitro* fermentation. The increase in *Prevotella* is linked to its ability to degrade fibers, particularly polysaccharides (60–62). *Phascolarctobacterium*, which is associated with SCFA production, has been shown to support host metabolic health (63), with its abundance significantly rising in the intestines of pigs fed high-fiber diets (64). This suggests that MCPC supports the growth of fiber-degrading bacteria by altering the gut environment. Additionally, the significant negative correlations between *Bacteroidota*, *Prevotella*, *Phascolarctobacterium,* and IVA reveal interactions between these microbes and intestinal metabolites. IVA is known for its role in protein fermentation, and it may influence microbial competition or fermentation pathways (65). The negative correlation indicates that MCPC may encourage the growth of fiber-degrading bacteria and reduce IVA production by shifting the fermentation of protein. This provides a deeper understanding of the interactions between microbes and metabolic products, reinforcing previous results. Furthermore, MCPC supplementation led to a reduction in the abundance of *Verrucomicrobiota*, *Actinobacteriota* and *Euryarchaeota* in the gut of growing pigs, with a trend toward an increase in *Spirochaetota*. Previous studies have shown that high concentrations of *Bifidobacterium* (Phylum: *Actinobacteria*) are positively correlated with the consumption of complex carbohydrates (66). In line with this, our study found the significant positive correlations between *Actinobacteria, Bifidobacterium* and IVA, IBA and/or VA. Therefore, the decrease in the abundance of these microbiota may be related to the reduced availability of complex carbohydrates (such as fibers) in the hindgut, likely due to MCPC’s effects on improving overall nutrient utilization. *Verrucomicrobiota*, known for carbohydrate degradation (67), may have its abundance reduced due to the inclusion of exogenous enzymes in the diet, which could reduce the need for endogenous carbohydrate-degrading enzymes in the gut. Additionally, MCPC supplementation significantly lowered the abundance of *Clostridium_sensu_stricto_1*, a key producer of microbial phytase (68), suggesting that without MCPC supplementation, growing pigs may not meet their phytase requirements, and the body may compensate by increasing reliance on microbial phytase to enhance nutrient digestion and absorption. *Streptococcus*, involved in lactic acid fermentation (69), may play an important role in acidifying the gut and inhibiting harmful bacteria. The observed trend toward increased relative abundance of *Streptococcus* in the MCPC group could support improved gut health and nutrient absorption. Overall, both *in vivo* and *in vitro* results indicate that MCPC supplementation improves the availability of fermentable substrates in the diet, promoting the growth of beneficial microbes. These microbial shifts optimize fermentation, particularly the breakdown of fibers and polysaccharides, thereby enhancing nutrient digestion efficiency and improving the utilization of less digestible substrates in the hindgut.

## 5 Conclusion

Supplementation with MCPC in the diet improves nutrient digestion and absorption in the foregut, while also supplying fermentable substrates that support microbial fermentation in the hindgut. It improves gut microbiota composition, promoting a balanced microbial ecosystem that enhances hindgut fermentation. This synergistic effect of foregut and hindgut improves the digestion of nutrients and the overall absorption and utilization efficiency, which is beneficial to animal growth and feed resource saving.

## Data Availability

The data presented in the study are deposited in the NCBI repository, accession number PRJNA1221116.
