# Supplementary material for: Synergistic effects of multi-enzyme supplementation on nutrient digestion and absorption in the foregut and hindgut
Source: Front Vet Sci. 2025 Feb 25;12:1554919. doi: 10.3389/fvets.2025.1554919 (PMC11893834; doi:10.3389/fvets.2025.1554919)
Supplement: Supplementary file 1 [file Table_1.docx]

**Synergistic Effects of Multi-Enzyme Supplementation on Nutrient Digestion and Absorption in the Foregut and Hindgut**

**Fangyuan Chen ^1,^†, Lianpeng Zhao ^1,^†, Lingjie Huang ^1^, Yong Zhuo ^1^, Shengyu Xu ^1^, Yan Lin ^1^, Lianqiang Che ^1^, Bin Feng ^1^ , De Wu ^1^, Zhengfeng Fang^1,2 *^**

^1^ Key Laboratory for Animal Disease Resistance Nutrition of the Ministry of Education, Animal Nutrition Institute, Sichuan Agricultural University, Chengdu, Sichuan 611130, China

^2^ College of Food Science, Sichuan Agricultural University, Yaan, Sichuan 611130, China

* Correspondence:

Zhengfeng Fang

[zfang@sicau.edu.cn](mailto:zfang@sicau.edu.cn).

†These authors contributed equally to this work.

Supplementary Material

Supplementary Table 1 Sequence of primers used for RT-qPCR

| Genes | Forward (5’-3’) | Reverse (5’-3’) |
| --- | --- | --- |
| *TRPV5* | AGGGTCGGTTTCTCTCGCTA | GGCATAGGTGATGGTGATGACA |
| *CaBP-D28K* | CGAGATCTGGCACCACTACG | ACCTGAGCAAGCTCAACGAT |
| *SLC17A4* | TTTTCAATTTCCACCCAACAAAT | GGGTGGGCAGAGCTGTGT |
| *SLC34A1* | TGGGCTTGTGTGACTGAGAG | CCCAGTCAGAGTTGTGCGTA |
| *SLC34A3* | TCGTCCTGGTCACAGTCC | CGGGGTTCTCATAGCAGTG |
| *SLC40A1* | GGAGCATCAGCTGTAACTGGAA | CCGAACCAGGCCACATTTT |
| *VDR* | TTGCCAAACACCTCAAGCACAAGG | TGCTCTACGCCAAGATGATCCAGA |
| *GLUT2* | GACACGTTTTGGGTGTTCCG | GAGGCTAGCAGATGCCGTAG |
| *FABP2* | TCGGGATGAAATGGTCCAGACT | TGTGTTCTGGGCTGTGCTCCA |
| *CD36* | GGAGAAAAGATCACTACCATCATGAG | CTCCTGAAGTGCAATGTACTGACA |
| *SLC1A4* | ACCCTCGCCGACTTTTAGTCT | GCCTGTGCCGAGAAGTAATCC |
| *PEPT1* | GGATAGCCTGTACCCCAAGCT | CATCCTCCACGTGCTTCTTGA |
| *β-actin* | GGATGACGATATTGCTGCGC | GATGCCTCTCTTGCTCTGGG |

Abbreviations: *TRPV5*, transient receptor potential cation channel subfamily V; *CaBP-D28K*, Calbindin-D28k; *SLC17A4*, solute carrier family 17 member 4; *SLC34A1*, sodium-dependent phosphate transporter 2A; *SLC34A3*, sodium-dependent phosphate transport protein 2C; *SLC40A1*, solute carrier family 40 member 1; *VDR*, vitamin D receptor; *GLUT2*, glucose transporter 2; *FABP2*, intestinal fatty acid binding protein; *CD36*, platelet glycoprotein 4; *SLC1A4*, solute carrier family 1 member 4; *PEPT1*, oligopeptide transporter 1.

Supplementary Table 2 Effects of MCPC on the digestibility of dietary gross energy and crude protein during *in vitro* enzymatic hydrolysis.

| Item | CON | MCPC | *P*-value |
| --- | --- | --- | --- |
| GE，% | |  |  |
| PC | 39.39 ± 1.48 | 38.84 ± 2.88 | 0.780 |
| NC1 | 37.77 ± 3.04 | 37.64 ± 2.36 | 0.958 |
| NC2 | 37.64 ± 1.24 | 37.49 ± 0.86 | 0.890 |
| CP，% | |  |  |
| PC | 53.94 ± 0.65 | 53.42 ± 2.51 | 0.740 |
| NC1 | 53.69 ± 0.73 | 53.29 ± 0.36 | 0.424 |
| NC2 | 54.73 ± 0.45 | 52.84 ± 3.07 | 0.334 |

MCPC = NSP enzymes and phytase complex. n = 4 / treatment.

Supplementary Table 3 Effects of MCPC on the content of pH value of the supernatant of dietary enzymatic hydrolysis products during *in vitro* fermentation

| Item | CON | MCPC | *P*-value |
| --- | --- | --- | --- |
| PC |  |  |  |
| 0 h | 8.06 ± 0.05 | 8.03 ± 0.05 | 0.523 |
| 48 h | 7.79 ± 0.06 | 7.80 ± 0.04 | 0.824 |
| 0-48 h | 0.27 ± 0.04 | 0.23 ± 0.09 | 0.547 |
| NC1 | |  |  |
| 0 h | 8.00 ± 0.06 | 7.97 ± 0.01 | 0.489 |
| 48 h | 7.80 ± 0.11 | 7.78 ± 0.05 | 0.697 |
| 0-48 h | 0.20 ± 0.07 | 0.20 ± 0.04 | 0.99 |
| NC2 | |  |  |
| 0 h | 8.06 ± 0.10 | 8.02 ± 0.02 | 0.439 |
| 48 h | 7.88 ± 0.07 | 7.84 ± 0.03 | 0.400 |
| 0-48 h | 0.19 ± 0.13 | 0.18 ± 0.04 | 0.901 |

MCPC = NSP enzymes and phytase complex. n = 4 / treatment.
